# Birth Preparedness and Complication Readiness among Pregnant Women in a Secondary Health Facility in Abakaliki, Ebonyi State, Nigeria

**DOI:** 10.1155/2020/9097415

**Published:** 2020-07-25

**Authors:** Chidebe Christian Anikwe, Bartholomew Chukwunonye Okorochukwu, Cyril Chijioke Ikeoha, Obiora G. K. Asiegbu, Ugochukwu Uzodimma Nnadozie, Justus Ndulue Eze, Johnson Akuma Obuna, Francis Chigozie Okoroafor

**Affiliations:** ^1^Alex Ekwueme Federal University Teaching Hospital Abakaliki Ebonyi state, Nigeria; ^2^Department of Obstetrics and Gynaecology, Federal Medical Centre, Owerri, Imo State, Nigeria

## Abstract

**Background:**

Birth preparedness and complication readiness (BP/CR) concept is based on the premise that preparing for birth and being ready for complications reduce all three phases of delay to a bad obstetric outcome.

**Objectives:**

To determine the knowledge of BP/CR with its determinants and BP/CR index among pregnant women in Abakaliki, southeast Nigeria.

**Methods:**

A cross-sectional survey was done between 1^st^ March 2019 and 31^st^ July 2019 among 450 randomly selected antenatal attendees at Mile Four Hospital, Abakaliki, Nigeria. The data were obtained using a pretested interviewer-administered structured questionnaire adapted from the maternal and neonatal health program handbook of the Johns Hopkins Program for International Education in Gynaecology and Obstetrics (JHPIEGO). The data obtained were analyzed using percentages, chi-square, and odds ratios. The level of significance is at *P* value < 0.05.

**Results:**

The birth preparedness and complication readiness index was 41.9%. Only 44.9% and 36.9% of the study population had adequate knowledge of birth preparedness (BP) and complication readiness (CR), respectively. Upper social class, lower educational level, urban residence, and less than 30 years of age were associated with increased odds of respondents having adequate knowledge of BP and CR (*P* > 0.05). However, only booking in the 1^st^ or 2^nd^ trimester was a significant determinant of the respondent's adequate knowledge of BP (AOR = 0.63, 95% CI 0.40-0.98) and CR (AOR = 0.62, 95% CI 0.39-0.97). Identification of transport and saving of money was the commonest birth plan while the commonest danger sign known to the participants was bleeding.

**Conclusion:**

This study revealed that knowledge of BP/CR is suboptimal. The determinant of this knowledge is antenatal booking. It is recommended that women should have adequate antenatal care education to improve their knowledge of BP/CR. This will help to increase the low BP/CR index seen in our study.

## 1. Introduction

Maternal death is a human tragedy and a violation of women's right to life [1, 2]. Globally, the maternal mortality ratio has been reported to be 216 per 100,000 deliveries with 303,000 maternal deaths occurring in 2015 [3]. The majority (99%) of these deaths occur in developing countries with sub-Saharan Africa accounting for more than 66% (201,000 maternal deaths) of the global estimate of developing countries [3, 4]. Nigeria is one of the eighteen countries in sub-Saharan Africa that has a very high maternal mortality ratio (MMR) in 2015 [3, 4]. It was estimated to be 814 (596–1180)/100,000 live births [4], and it is higher in areas where there is poor access to skilled birth attendants [5]. Studies done in Nigeria have reported MMR of 532/100,000 in Ilorin [6], 2735.6 in Port Harcourt [7], 2151 in Sokoto [8], 772 in Enugu [9], 1359 in Abakaliki [10], and 2420 in Kano [11].

In Nigeria, the majority of these deaths occur during labor and in the postpartum period [12] with the variation of the leading causes in different regions of the country [10, 13–17]. Access to antenatal care during pregnancy, skilled care during childbirth, and the puerperal period are important in reducing this maternal wastage [3, 4]. Apart from the biomedical causes of maternal deaths, several other risk factors predispose to the high number of women that die during childbirth. This is opined in the three-delay model by Thaddeus and Maine [18]. Thaddeus and Maine [18] identify delays in seeking, reaching, and obtaining requisite care as the key factor leading to maternal death.

Birth preparedness and complication readiness (BP/CR) concept is a global strategy in reducing maternal mortality. It is based on the premise that preparing for birth and being ready for complications reduce all three phases of delays in receiving services by skilled birth attendants [19], (Table 1). It is a matrix of shared responsibilities involving the pregnant woman/family, community, hospital facility/provider, and policymakers [19]. Since normal pregnancy, labor, and puerperium are a retrospective diagnosis, the concept of BP/CR helps each group in advance to proactively prepare for the ultimate goals of the safe childbirth process. Studies done in Nigeria have highlighted the contribution of these delays to maternal death [20, 21]. Type I and III delays were responsible for 35.5% and 48.48% of maternal deaths in Calabar [20] while in Benin City, it accounted for 28.6% and 61.9% of maternal deaths [21]. It is quite worrisome of the contribution of type III delays to maternal death. Igwegbe et al. [22] have shown that improvement in service delivery in the hospital could help reduce maternal deaths attributable to type III delays. Reports have shown that pregnant women in Nigeria are poorly prepared to face challenges that might occur during the childbirth process. In Ife, Nigeria, Kuteyi et al. [23] reported that only 34.9% of women studied were prepared for childbirth while it was less than 7% in a community-based study in Northern Nigeria [24]. These findings agree with reports from regions of Ethiopia [25–27] and Chamwino district of Tanzania [28]. Women's level of education; antenatal care attendance; previous obstetric outcome; knowledge of BP/CP plans; place of residence; parturient knowledge of key danger signs of pregnancy, of labor, and in the postpartum period; maternal age; parity; and marital status were some of the factors influencing parturient preparedness for childbirth [26, 27, 29, 30]. It thus emphasizes the importance of individual and community preparation for reducing bad childbirth outcomes.

A low level of BP/CR among antenatal attendees might be one of the factors contributing to the high maternal mortality rate in our region. A systematic review has shown the effectiveness of BP/CR in reducing bad obstetric outcomes in low-resource settings like sub-Saharan Africa [31], thus emphasizing its advocacy in Nigeria. In Abakaliki, the MMR is unacceptably high [10] with the majority of these women presenting very late to the hospital, thus highlighting the need of educating our obstetric population on the need for BP/CR. It has been argued that the improvement of expectant mother's knowledge about BP/CR strategy is associated with the use of skilled birth attendants (SBA) during pregnancy and delivery [32] with its attendant benefits. It, therefore, becomes imperative that knowledge of BP/CR should be assessed among expectant mothers in the area; the finding would assist in the counseling of these women and development of strategies towards the improvement of maternal and child health outcomes in Abakaliki, Nigeria. It will help also to generate a cross talk between the stakeholders of fetomaternal wellbeing in the state on the need to invest more in human and infrastructural development needed for safe motherhood. All these are hoped will assist in reducing the burden of maternal and fetal morbidity and mortality in the study area through the uptake of skilled birth care. The current study is aimed at determining the knowledge of birth preparedness and complication readiness and its determinants among pregnant women in a secondary health facility in Abakaliki, southeast Nigeria. It will also help to determine the overall BP/CR index of the study population. This index will indirectly assist us in auditing our antenatal care services through the identification of areas of focus in our antenatal classes.

## 2. Materials and Methods

### 2.1. Study Design

This study is a cross-sectional study.

### 2.2. Study Area

This cross-sectional study was carried out at St. Patrick's Mile Four Hospital, Abakaliki, one of the mission hospitals in the state. It was established in 1964 by late Bishop McGettrick to care essentially initially for pregnant leprosy patients but later extended their care to maternal and child health services. It has an average of 330.8 deliveries per month, and it receives a referral from primary health centres and general hospitals. It has an operation room staffed 24 hours a day by nurses and physicians capable of performing cesarean sections, trained midwives, and good blood banking services.

Ebonyi State is one of the states in southeast Nigeria with a total population of 2,176, 947 million in 2018, and a total landmass of 5,533 km^2^. The main occupation of the people is farming. It shares a boundary with the following states in Nigeria: Enugu, Cross River, Abia, and Benue. The state has 13 local governments with one urban, 2 semiurban, and others are rural. Abakaliki is the capital city of Ebonyi State and located 64 kilometers southeast of Enugu. In Ebonyi State, government and nongovernmental agencies encourage women to deliver in hospitals manned by skilled birth attendants to avoid home deliveries or deliveries by a traditional birth attendant (TBA). They provide transport services to the rural obstetric populace, community health extension workers (CHEW), and free antenatal care and delivery services in partnership with the various mission and government-owned hospitals in the state.

### 2.3. Study Population

#### 2.3.1. Inclusion Criteria

Women included in the study were those that have achieved gestational age of 36 weeks and above, gave consent to participate, booked patients, and had at least three antenatal care visits.

### 2.4. Exclusion Criteria

Women excluded from the study were pregnant women who are not residents in the study area and those who were sick and incapable of being interviewed.

Women who were booked refer to women who had attended at least three visits in the hospital and had done their baseline booking investigations; the results of their investigation had been reviewed by a doctor. The baseline booking investigation was pack cell volume (PCV), blood group, genotype, retroviral screening, hepatitis B virus screening, venereal research laboratory test (VDRL), and urinalysis. They were given a health talk which usually covers various topical issues including nutrition, diet, personal hygiene, and danger signs in pregnancy, labor experience, care of the newborn, exclusive breastfeeding, and immunization. Other health issues such as hypertension, diabetes mellitus, malaria, anaemia, human immunodeficiency virus/acquired immunodeficiency syndrome (HIV/AIDS), and family planning were also discussed.

### 2.5. Sample Size and Sampling Technique

The sample size (*N*) was calculated using the following:

(1) $$\begin{array}{c} N=Z^{2}\frac{pq}{e^{2}}, \end{array}$$

where *Z*^2^ is a constant = 1.96; *e* is the desired level of precision, also known as sampling error: 5%; *p* is the estimated prevalence = 0.50; *q* is 1 − *p*; N = (1.96)^2^ × 0.50 × 0.50/(0.05^2^) = 384.16.

The attrition rate of 10% was added to the sample size, i.e., 384.16 + 38.41 = 422. The sample size of 450 was however used for the study to increase the power.

Those who met the inclusion criteria were selected using a ballot method of simple random sampling in the antenatal clinic after the health talk. The study population was recruited by using a ballot method of simple random sampling method after verbal consent was obtained. Consented women were asked to pick a piece of white cardboard paper marked Yes (include) and No (exclude) with replacement from a black polythene bag into which an equal number of cards. Yes and No cards were added. Women that picked a card marked Yes were recruited as the study population. The process was continued until the total sample size was gotten.

### 2.6. Data Collection

They were recruited between 1^st^ March 2019 and 31^st^ July 2019. They were interviewed using a structured questionnaire adapted from the Johns Hopkins Program for International Education in Gynaecology and Obstetrics- (JHPIEGO-) maternal and neonatal health program handbook. Pretesting of the questionnaire was done among 100 pregnant women selected at random in the health facility on different antenatal clinic days (20 questionnaires per day). The clarity and understanding of questions were ascertained, and changes were made based on their responses to improve the study instrument. Cronbach's alpha was 0.85. The data was collected by trained two senior registrars from the department and led by one of the authors. This was done before they were called in by the nurses to see their doctors. Consenting women who were literate filled the questionnaire while those who were not literate were interviewed alone in one of the offices dedicated to the study by the trained research assistants. The questionnaires were filled based on their responses. The filled questionnaires were cross-checked at the site for completeness before they were accepted, and those not properly filled were discarded. The social class of the study population was determined based on the social class classification of Olusanya et al. [33]. The educational level of the women and the occupation of the husband were used in the classification. The educational status of the respondents was grouped into tertiary (score = 0), secondary (score = 1), and primary/none (score = 2) while the husband's occupation was grouped into professional (score = 1), skilled (score = 2), and unskilled (score = 3). Various combinations of these scores were used to determine the social class of the respondent. The social class of the study participants that were not married was based on that of their mothers. They were graded into social classes 1 to 5. Social classes 1 and 2 were grouped as upper social class, social class 3 as middle class, and social classes 4 and 5 as lower social class.

### 2.7. Measurement

#### 2.7.1. Birth Preparedness and Complication Readiness

Birth preparedness and complication readiness (BP/CR) index is a set of indicators for monitoring of safe motherhood programs. It operates at six (6) levels which are the individual woman, her family (husband/partner), the community, the health facility, the provider, and the policymaker. BP/CR index for each item was calculated from the ratio of the number of the respondent's responses to the number of respondents sampled (see Table 2; for example, 141/450 = 31.3). The final score is simply the mean of the percentages for each item on the index (419.8/10 = 41.9).

#### 2.7.2. Knowledge of Danger Sign Assessment

They were assessed to have adequate knowledge of danger signs (for each section during pregnancy, labor, puerperium, and newborn) if she spontaneously mentions a total of three danger signs during pregnancy, four danger signs during labor/childbirth, three danger signs during the puerperium, and three danger signs in the newborn.

### 2.8. Knowledge of BP/CR Assessment

#### 2.8.1. Criteria for Assessment of Knowledge of Birth Preparedness

A respondent was assessed to have adequate knowledge of birth preparedness if she spontaneously mentioned at least 4 or more of the items as follows: place of delivery, saving money, arrangement for transportation, purchase or arrangement for the material, knew expected date of delivery (EDD), identify a decision maker/birth companion, and undergone human immunodeficiency virus (HIV) counseling and testing.

#### 2.8.2. Criteria for Assessment of Knowledge of Complication Readiness

A respondent was assessed to have adequate knowledge of complication readiness if she spontaneously mentioned at least 4 or more of the items as follows: adequate knowledge of danger signs, identify a hospital for delivery, identify a skilled birth attendant for delivery, saved or saving money, arrangement for transportation, and arrangement for blood.

#### 2.8.3. Data Analysis

The data was collected in a spreadsheet of our personal computer and analyzed using IBM SPSS Statistics version 20 (IBM Corp., Armonk, NY, USA). Frequency distribution was used to describe the background characteristics of the respondents. Pearson's chi-square test and logistic regression analysis were used to examine the association between sociodemographic/obstetric characteristics of the study population and knowledge of danger signs and knowledge of birth preparedness/complication readiness. The odds ratios with their 95% CI were calculated to determine the strength and presence of an association. A *P* value of ≤0.05 was considered significant. Odds ratio (OR) < 1 infers that persons in that category have a lower likelihood of knowledge of birth preparedness and complication readiness plan, while OR > 1 was designated increased probability of knowledge of birth preparedness and complication readiness plan.

#### 2.8.4. Ethical Consideration

Ethical approval for the study was obtained from the Mile 4 Research and Ethics committee. The ethical approval number is RE/M4H/29/19. Written consent was obtained from the participant before the administration of the questionnaire. For those that had no formal education, the consent forms were read out to them in the Igbo language (the study population was all Igbos) and the study was explained to them, making sure they fully understood the objectives of the study. The literate study population was allowed to read and sign the consent form while the illiterate ones either signed or used their thumbprint. They were assured that refusal to participate or their responses would not affect their care at the facility. All the interviews were conducted in complete privacy, and data collection tools were strictly anonymous.

## 3. Results

**Figure 1:**
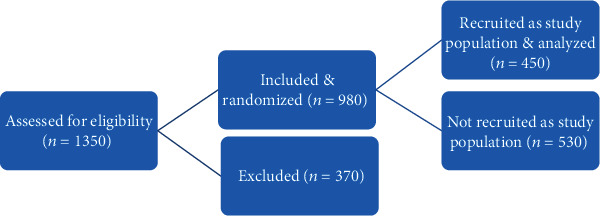


Over the study period of 5 months, 1350 patients were assessed for eligibility as documented in the inclusion and exclusion criteria above. Nine hundred and eighty women (980) met the inclusion criteria and were selected for randomization. Three hundred and seventy (370) were not eligible and were excluded. Out of 980 eligible women that were subjected to the ballot method of simple random sampling method, only four hundred and fifty women (450) were selected as the study population. The data were correctly collated and analyzed as summarized in Figure 1.

Table 3 represents the sociodemographic characteristics of the study population. All the women interviewed were included in the final analysis, giving a response rate of 100%. The mean age of the respondents was 29.3 ± 4.18 years with minimum and maximum age of 18 years and 40 years, respectively. About three quarters (335; 74.4%) of the women were urban dwellers and were mainly Christians. The majority (252; 56.0%) of the respondents had tertiary education as their highest level of education. The majority (171; 38.0%) of the respondents belonged to lower social classes.

Table 4 represents the obstetric characteristics of the study population. The respondent mean gestational age was 38.3 (95% CI 37.7–38.9) weeks. The respondents were mainly primiparous (181; 40.2%), and the mean parity was 1.75 ± 1.73. The entire respondents were booked with a mean gestational age at booking of 18.7 ± 6.93 weeks. The majority of the respondents (286; 63.1%) have attended 3.9 ± 2.35 antenatal visits before being recruited into the study.

As shown in Table 5, the entire respondent was aware of at least one danger sign in pregnancy, labor and childbirth, puerperium, and newborn, with 43.8% of the respondents having adequate knowledge. Vaginal bleeding was the most common danger sign known by the respondents during pregnancy (172, 38.2%) and labor (152; 33.8%) and in the postpartum period (164; 34.4%). In pregnancy, 21.6% of the respondents were aware that severe headache is a danger sign. More than 23.1% of the respondents were aware that labor lasting more than 12 hours is abnormal. Only 16.4% of the respondents were of the view that the inability to deliver the placenta within 30 minutes of delivery of the baby is a danger sign. In the newborn, yellowness of the skin/eye (134; 29.8%) and difficult breathing (107; 23.8%) were the most common problems known to the respondents. Only 40.4%, 39.1%, 37.3%, and 30.6% of the respondents had adequate knowledge of danger signs in pregnancy, childbirth, postpartum, and newborn period, respectively. Social class and gestational age at booking were significantly associated with the respondent's adequate knowledge of danger sign during pregnancy (*X*^2^ = 50.57 (24) *P* = 0.001; *X*^2^ = 64.78 (24) *P* = 0.001, respectively), labor (*X*^2^ = 31.89 (14) *P* = 0.001; *X*^2^ = 23.96 (14) *P* = 0.046, respectively), and puerperium (*X*^2^ = 57.47 (20) *P* = 0.001; *X*^2^ = 50.17 (20) *P* = 0.001, respectively) and in the newborn (*X*^2^ = 44.75 (18) *P* = 0.001; *X*^2^ = 30.16 (18) *P* = 0.036, respectively) (not in the table).

From Table 6, 44.9% of the study population had adequate knowledge of birth preparedness (BP). Women with less than tertiary education had increased odds of having adequate knowledge about birth preparedness when compared with a cohort with tertiary education. Logistic regression was performed to ascertain the effect of maternal age, level of education, place of residence, social class, gestational age, trimester of booking, and parity on the determinant of adequate knowledge of birth preparedness and complication readiness among the study population. The evaluation showed that only the trimester of booking and women's parity are the significant determinants of adequate knowledge of birth preparedness.

From Table 7, only 36.9% had adequate knowledge of complication readiness (CR). Booking in the 1^st^ or 2^nd^ trimester of pregnancy is associated with a 62% chance of having adequate knowledge of CR with a true population effect between 38% and 3% (*P* = 0.037). Women whose level of education is less than tertiary education have less chance of 31% of not being CR when compared with those with tertiary education with a true population effect that is between 89% and 46%, and it is statistically significant.

The birth preparedness and complication readiness index for the respondent is shown in Table 2. The knowledge of key danger signs is low, ranging between 26% and 32%. On the intention and behavior of the respondents, all the respondents are planning to have their delivery with a skilled birth attendant (SBA). Less than one percent (0.89%) of the respondents are making arrangements for blood if needed during their delivery. On the issue of awareness of any community provision for safe delivery, none of the women are aware of such provision.

## 4. Discussion

Birth preparedness and complication readiness is an important tool for safe motherhood. It encourages proactive, good health-seeking behaviors among expectant mothers which are important in preventing bad obstetric outcomes. In our study, only 44.9% and 36.9% of the respondents have adequate knowledge of birth preparedness and complication readiness, respectively. The commonest preparations made by the women were saving money, identification of birthplace, and making arrangements for transportation. This is in concordance with findings in Nigeria [23, 34] and the Mbarara district of Uganda [35]. The percentage of women who are birth-prepared in our study is, however, higher than the findings of Kuteyi et al. (34.9%) [23], Debelew et al. (34.5%) [27], Asrat et al. (27.5%) [29], and Hailu et al. (17.0%) [36]. It is, however, much lower than the 87.4% reported by Tobin et al. in Nigeria [34] and 82.3% reported by Urassa et al. [37] in Tanzania. Differences in the study population could be responsible for the above differences from our study. Our study revealed that belonging in upper social class, urban residence, booking in the first or second trimester, and duration of pregnancy less than 30 weeks are associated with an increased likelihood of having adequate knowledge of birth preparedness. This is expected as the above factors provide windows of opportunity for an expecting mother to be educated during pregnancy [29]. In our study, the place of residence is not a significant contributor to women's adequate knowledge of birth preparedness (OR = 1.35; 95% CI 0.88-2.08, *P* = 0.166) which is in keeping with an earlier study by Ekabua et al. in southeast Nigeria [30]. Our finding, however, agrees with the work of Asrat et al. [29] that reported that urban dwellers were more knowledgeable of birth preparedness.

The level of education of a woman is expected to influence her behavior on issues of health matter [38]. In bivariate analysis, this association was seen as women with higher education (OR = 1.13; 95% CI 0.89-1.44) have an increased likelihood of being BP. This advantage was however lost in the multivariate analysis where the converse is true, although not significant (OR = 1.23; 95% CI 0.84-1.81). A likely explanation to the above finding is that women with lesser educational attainment and probably in lower social class are prepared because of their seemingly disadvantaged position while the educated (women with tertiary education) are complacent unlike what is expected from previous reports [30, 37]. The influence of the place of residence on BP is likely to be the effect of the upper social class, as women who are in the upper social class are expected to reside in urban areas. From the study, booking early with antenatal follow-up visits and being nulliparous or primiparous women are the significant determinants of BP. This is keeping with various reports on this issue [27, 29, 39]. It is, therefore, important that women in Abakaliki and by extension Nigeria, in general, should be encouraged to book early in pregnancy to improve their knowledge on BP/CR. This is hoped will assist in preparing our obstetric population to face the challenges of being pregnant and delivery can pose. On the issue of parity, it should be expected that multiparous women should be more knowledgeable of BP than those that are primiparous or nulliparous, but the reverse was our finding. This lack of knowledge by the multiparous women can be due to the lack of proper information on BP/CR from their previous antenatal classes as the majority of the maternity hospitals in the state (Abakaliki, Ebonyi State) are still practicing the traditional method of antenatal care that emphasizes on quantity and not on the quality of care [40].

Normal pregnancy and childbirth are a retrospective diagnosis that calls for emergency preparation. Planning for this, by all the players involved, hastens early maternal access to the use of skilled services during complications [29]. Expectant mother's adequate knowledge of complication readiness is essential to the attainment of the delivery of a healthy baby to a healthy and satisfied mother. The index study shows that less than two-fifths of the women have adequate knowledge of complication readiness, unlike in the Kuteyi et al. [23] study where more than half (66.1%) of their study population are complication ready. Our low value is worrisome and might be a reflection of the general expectation by the expecting mothers in the study area that their pregnancy would end well, not aware that normal pregnancy and labor are retrospective diagnoses. Lack of adequate information from the organizers of antenatal classes might also account for this low level of knowledge. It is therefore paramount that a concerted effort should be made to adequately inform the antenatal attendees in the study area of its importance. The significant determinants of CR in our study were the trimester of booking and the level of education. This could be attributed to reason earlier adduced above for BP. Women whose duration of pregnancy at booking is below 30 weeks are more likely to have adequate knowledge about complication readiness (OR = 0.71; 95% CI 0.48-1.05) as more information about the concept (BP/CR) is expected to be gotten as pregnancy advances. This is in agreement with the previous studies [34, 41, 42].

The birth preparedness and complication readiness index (BP/CR index) in our study is 41.9% which is comparable to the 44.15% reported in one antenatal attendee population in the southeast of Nigeria [43]. The BP/CR matrix can be used in a variety of ways like to demonstrate and support shared responsibility and accountability for safe motherhood. Using the matrix, advocacy groups can reach stakeholders, program planners, and policymakers to generate cross talk on issues of safe motherhood. These will help to mobilize the necessary human and fiscal resources to identify barriers and solutions proffered to the elimination of delays to maternal death. The percentage of women who are making arrangements to provide blood was less than one percent in our study and is very low compared with other studies [37, 43, 44]. Possible reasons for nonprovision of blood might include fear of blood transfusion, wrong belief that provision of blood means wishing for a complication like postpartum hemorrhage, or cesarean section which is dreaded in the study area [45]. The BP/CR index gives an objective measure of the client's knowledge of key danger signs, her intention and behavior, and her knowledge of community resources for safe motherhood [19]. The index being less than fifty percent in the study population may be one of the factors responsible for the high burden of maternal [10] wastage in the area of study. It is revealed that none of the study population is aware of any community resources for safe motherhood; this is unlike the finding from a neighboring state where some percentage of the women is aware, although insignificant, of the availability of such resources [43]. Our findings might be explained by a lack of knowledge as seen above but could also result from the nonexistence of any community resources aimed at assisting pregnant women in the study area.

There is a good awareness of danger signs among the study population although only 40.4%, 39.1%, 37.3%, and 30.6% of the respondents had adequate knowledge of danger signs in pregnancy, childbirth, postpartum, and newborn period, respectively. The commonest danger sign known is vaginal bleeding, which is in keeping with earlier studies by Igwegbe et al. [22], Ekabua et al. [30], Agunwa et al. [46], Tilahun and Sinaga [47], Oni et al. [48], Urassa et al. [37], and Iliyasu et al. [24] that reported that vaginal bleeding is the commonest danger sign known to the women in their respective studies. This might be expected as vaginal bleeding outside menstruation connotes danger and is likely to elicit a prompt response in a woman. The social class of the women and gestational age at booking are significantly associated with respondent knowledge of danger signs. Support on this finding was seen in the work of Maroof et al. [49], Ibadin et al. [50], and Tobin et al. [34] that reported on the contribution of the level of education of women, women's occupation, antenatal care, being married, and husband's income on a client being birth-prepared and complication ready. The influence of gestational age at booking might be attributed to an increase in the acquisition of knowledge of danger signs from antenatal classes. This highlights the importance of advocacy for early booking of pregnancy with a skilled birth attendant as a tool in reducing maternal and fetal mortality and morbidity in Nigeria.

### 4.1. Limitations of the Study

The terms “adequate knowledge” and “complication ready/birth-prepared” as used to express the authors' assessment of knowledge of danger signs in pregnancy and BP/CR practices are discretionary because of lack of uniform standards. The meaning of these terms may, therefore, vary significantly from the previous usage in other similar literature. Another limitation of the study is that it is not possible to determine causal relationships but only to test for associations because of the cross-sectional nature of the study. There may have been recall bias, and some respondents may have been reluctant to disclose information as they might view our interview as an audit of care they are receiving from the hospital. They might fear a possible retributive action from the management. An effort was, however, made to avoid this by interviewing them in a dedicated office and making sure that their identity is not on the questionnaire. The finding in our study might be influenced by the tool used in accessing it. An effort was, however, made to reduce these errors by educating the respondent on the study instrument and pretesting the instrument before applying it in the field.

### 4.2. Strength of the Study

The group of pregnant women studied was women at term which are expected to enter into labor, and the index study highlighted how proactively they are to face the process of childbirth. This could be a reflection of the general obstetric population in the study area as a significant number of pregnant women in the study area patronize Mile Four Hospital for delivery. Our study provides useful information that could guide hospital managers and policymakers on how to improve care and reduce maternal and neonatal morbidity and mortality in Abakaliki and Nigeria in general. Even though it is a hospital-based study, the relatively large number of the women studied and the random selection of the study population assisted in increasing the power of our study. The instrument used in our study was derived from JHPIEGO and was validated before it was administered to the participant.

## 5. Conclusion

The current study has shown that the level of knowledge of birth preparedness and complications among our study population is worrisomely low. As every pregnancy is prone to complications, the level of complication readiness should be improved upon through education of the obstetric population. Our study shows that antenatal booking is a significant determinant of adequate knowledge of birth preparedness and complication readiness. Health managers in Abakaliki, Ebonyi State, Nigeria, should disseminate information to the obstetric population on the importance of early booking with a skilled birth attendant as this will provide a veritable opportunity to educate pregnant women on the concept of BP/CR. In the long run, the educational level of our girl, child, and current obstetric population must be improved upon as this is shown to contribute to increasing knowledge. There is also a need for the Ebonyi State government and nongovernmental agencies (NGOs) to assist the government in providing enabling resources for safe motherhood at the community level as this is found to be deficient or unavailable in our study.

## 6. Recommendations

We recommend that early booking should be encouraged in the study area and adequate time devoted to educating pregnant women on the danger signs of pregnancy and the concept of BP/CR during antenatal care by health professionals. It is important that the social class of women is improved in the study area via women empowerment, as this contributes significantly to the knowledge of danger signs in pregnancy. The community health extension worker (CHEW) should be employed by the government to help improve the knowledge and practices of antenatal women during their community visit.

## Figures and Tables

**Table 1:** Danger signs.

(1) Pregnancy	Vaginal bleeding, swollen hands/face, blurring of vision, convulsion, high fever, reduced fetal movements, severe weakness, liquor drainage without labor
(2) Labor and childbirth	Labor lasting >12 hours, convulsion, inability to deliver placenta > 30 minutes after delivery, severe vaginal bleeding, severe headache, high fever, blurred vision, difficulty in breathing, severe weakness, loss of consciousness and swollen hands/face
(3) Puerperium—during the 1^st^ second-day postdelivery	Profuse vaginal bleeding, high fever, offensive vaginal discharge, convulsion, loss of consciousness, severe weakness, difficulty in breathing
(4) Newborn—during the 1^st^ seven days after birth	Difficult or fast breathing, yellow skin/eye colour, pus, bleeding or discharge from/around the umbilical cord, baby very small, red or swollen eyes with pus and unconsciousness

**Table 2:** Birth preparedness and complication index (BP/CR index) for the study population.

Variable	Frequency (*n*)	Percentage (%)
Knowledge of key danger sign		
% of women who know danger sign during pregnancy	141	31.3
% of women who know danger sign during labor	138	30.7
% of women who know danger sign during postpartum	134	29.8
% of women who know danger sign in newborn	119	26.4
Service use and planning actions: intention and behavior		
% of women who plan to attend at least 4 ANC with a SBA	450	100.0
% of women who plan to give birth with a SBA	450	100.0
% of women who attended first ANC with a SBA during the first trimester	97	21.6
% of women who plan to save money for childbirth	219	48.7
% of women who plan to identify a mode of transport to the place of childbirth	133	29.6
% of women who plan to arrange blood	4	0.89
Knowledge of community resources		
% of women who knew that their community have a financial support system	0	0
% of women who knew that their community has a transportation system	0	0
% of women who knew that their community has a blood donor system	0	0
Total	1885	418.99
BP/CR index for the study population		41.90

SBA: skilled birth attendant; ANC: antenatal care.

**Table 3:** Sociodemographic characteristics of the study population.

Maternal variable	Frequency (%)
Age	
≤20	9 (2.0)
21-25	68 (15.0)
26-30	198 (44.0)
31-35	153 (34.0)
>35	22 (5.0)
Residence	
Urban	335 (74.4)
Rural	115 (25.6)
Religion	
Christianity	446 (99.1)
Others	4 (0.9)
Ethnicity	
Igbo	450 (100.0)
Others	—
Educational status	
No formal education	11 (2.4)
Primary	41 (9.1)
Secondary	146 (32.5)
Tertiary	252 (56.0)
Social class	
Upper class	128 (28.0)
Middle class	151 (34.0)
Lower class	171 (38.0)

**Table 4:** Obstetric characteristics of the study population.

Maternal variable	Frequency (%)
Bookings	
First trimester	106 (23.6)
Second trimester	297 (66.0)
Third trimester	47 (10.4)
Parity	
Nullipara	137 (30.4)
Primipara	181 (40.2)
Multipara	132 (29.4)
Gestational age at booking	
≤13	11 (2.5)
14-28	122 (27.1)
≥29	317 (70.4)
No. of ANC visit	
≤4	286 (63.1)
>4	166 (36.9)

ANC: antenatal care.

**Table 5:** Awareness of danger signs during pregnancy, labor, and puerperium and in newborn^∗^.

Danger sign	Frequency (%)
Pregnancy	
Bleeding	172 (38.2)
Severe headache	97 (21.6)
Blurred vision	54 (12.0)
Convulsion	66 (14.7)
Swollen hands/face	78 (17.3)
High fever	93 (20.7)
Loss of consciousness	50 (11.1)
Difficulty breathing	37 (8.2)
Severe weakness	58 (12.9)
Severe abdominal pain	64 (14.2)
Reduced fetal movement	45 (10.0)
Drainage of liquor	67 (14.9)
Labor	
Bleeding	152 (33.8)
Severe headache	81 (18.0)
Convulsion	58 (12.9)
High fever	74 (16.4)
Loss of consciousness	55 (12.2)
Labor lasting >12 hours	104 (23.1)
Placenta not delivered after 30 minutes	74 (16.4)
Puerperium	
Bleeding	164 (34.4)
Severe headache	91 (20.2)
Blurred vision	44 (9.8)
Convulsion	57 (12.7)
Swollen hands/face	45 (10.0)
High fever	79 (17.6)
Offensive vaginal discharge	56 (12.4)
Loss of consciousness	39 (8.7)
Difficulty breathing	41 (9.1)
Severe weakness	43 (9.6)
Newborn	
Difficulty breathing	107 (23.8)
Yellow skin/eye	134 (29.8)
Poor feeding	101 (22.4)
Abnormal discharge from umbilical cord	65 (14.4)
Baby very small	45 (10.0)
Skin blisters	44 (9.6)
Convulsion	75 (16.7)
Unconsciousness	44 (9.8)
Red swollen eye	43 (9.6)

^∗^Multiple answers allowed.

**Table 6:** Cross-tabulation of sociodemographic and obstetric characteristics of the study population with knowledge of birth preparedness.

Maternal variable	Birth preparedness		AOR (95% CI), *P* value	
	Yes	No		
Age				
≤30 years	119	153	1.11 (0.76-1.63)	0.561
>30 years	83	95	1.00	
Education				
Below tertiary	129	146	1.23 (0.84-1.81)	0.273
Tertiary	74	102	1.00	
Residence				
Urban	157	178	1.35 (0.88-2.08)	0.166
Rural	45	70	1.00	
Social class				
Upper class	66	75	1.13 (0.75-1.68)	0.542
Below upper class	136	173	1.00	
GA at booking				
≤30 weeks	111	116	1.36 (0.94-1.98)	0.102
>30weeks	91	132	1.00	
Trimester of booking				
1^st^ and 2^nd^	147	200	0.63 (0.40-0.98)	0.044
3^rd^	55	48	1.00	
Parity				
0-1	131	139	1.45 (0.99-2.12)	0.057
2 and above	71	109	1.00	

GA: gestational age.

**Table 7:** Cross-tabulation of sociodemographic and obstetric characteristics of the study population with knowledge of complication readiness.

Maternal variable	Complication readiness		AOR (95% CI), *P* value	
	Yes	No		
Age				
≤30 years	101	171	0.97 (0.65-1.44)	0.895
>30 years	65	113	1.00	
Education				
Below tertiary	114	160	1.69 (1.13-2.54)	0.010
Tertiary	52	124	1.00	
Residence				
Urban	121	214	0.88 (0.56-1.36)	0.564
Rural	45	70	1.00	
Social class				
Upper class	55	86	1.14 (0.75-1.72)	0.529
Below upper class	111	198	1.00	
GA at booking				
≤30 weeks	74	151	0.71 (0.48-1.05)	0.092
>30 weeks	92	133	1.00	
Trimester of booking				
1^st^ and 2^nd^	119	228	0.62 (0.39-0.97)	0.037
3^rd^	47	56	1.00	
Parity				
0 to 1	107	163	1.34 (0.90-1.99)	0.141
2 and above	59	121	1.00	

GA: gestational age.

## Data Availability

All data generated or analyzed during this study are included in this published article.

## Abbreviations

ANC: Antenatal clinic
BP: Birth preparedness
CR: Complication readiness
BP/CR: Birth preparedness and complication readiness
CHEW: Community health extension worker
JHPIEGO: Johns Hopkins Program for International Education in Gynaecology and Obstetrics
HIV/AIDS: Human immunodeficiency virus/acquired immunodeficiency syndrome
MMR: Maternal mortality ratio
NGOs: Nongovernmental agencies
SBA: Skilled birth attendant
STIs/PMTCT-plus: Sexually transmitted infections and prevention of mother-to-child transmission of HIV
TBA: Traditional birth attendant
VDRL: Venereal research laboratory test.
